# Intimate partner violence as a predictor of antenatal care services utilization in Rwanda

**DOI:** 10.1186/s12884-021-04230-5

**Published:** 2021-11-08

**Authors:** Claire Bahati, Josias Izabayo, Japhet Niyonsenga, Vincent Sezibera, Léon Mutesa

**Affiliations:** 1 Department of Clinical Psychology, College of Medicine and Health Sciences, University of Rwanda, Kigali, Rwanda; 2 Centre for Mental Health, College of Medicine and Health Sciences, University of Rwanda, Kigali, Rwanda; 3 Mental Health & Behaviour Research Group, College of Medicine and Health Sciences, University of Rwanda, Kigali, Rwanda; 4 Centre of Human Genetics, College of Medicine and Health Sciences, University of Rwanda, Kigali, Rwanda

**Keywords:** Intimate partner violence, Antenatal care, Pregnant women, Reproductive health, Rwanda

## Abstract

**Background:**

Although compelling evidence shows that exposure to intimate partner violence (IPV) during pregnancy is detrimental to both physical and mental health of the victims and their fetuses, studies on negative impact of IPV on antenatal care (ANC) services utilization are scarce.

**Methods:**

The aim of the current study was to determine the impact of IPV exposure on ANC services utilization indicators such as (i) initiation of care within the first 3 months of pregnancy, (ii) receipt of at least four ANC visits and (iii) receipt of care from skilled providers among reproductive age women in Rwanda. This study used the data from the 2014–15 Rwanda Demographic and Health Survey. Multiple logistic regression was used to estimate the effects of physical and sexual IPV on the ANC services utilization indicators.

**Results:**

Among married women living with their partners with at least one child aged 5 years or under (*N* = 5116), 17% of them reported physical violence, 22.8% reported psychological violence and 9.2% reported sexual violence. We found that there was a significant negative relationship between physical IPV and both early ANC and sufficient ANC. Women who had experienced physical violence by their partners during the preceding 12 months were less likely to receive more than four ANC visits, (O.R = 0.61, CI = 0.417–0.908) and they were less likely to attend the first ANC visits within the first 3 months (O.R = 0.656, CI = 0.445–0.967).

**Conclusion:**

In this study, the prevalence of IPV still remains high and there is evidence that it does have significant impact on ANC. Therefore, the results provide support for continued efforts to reduce intimate partner violence, through the improvement of screening for IPV during ANC visits.

## Background

Intimate partner violence (IPV) refers to behavior within an intimate relationship that causes physical, psychological or sexual harm to those in the relationship [1]. Intimate partner violence is a global public health issue that affects about one third of women globally [2]. Global estimates of intimate partner violence perpetuated by men against women indicate that 30% of every partnered women worldwide have experienced physical and/or sexual violence by an intimate partner at some point in their lifetime [3]. The evidence shows that the overall prevalence rate of IPV during pregnancy in developed countries ranges between 10 and 20% [4]. Among developing nations, African countries have some of the highest rates of IPV during pregnancy with Gambia leading at 61%; followed by Zimbabwe (34%); Kenya, where it is estimated that 38% of women suffer from IPV in their lifetime [3]; and Ethiopia (25%) [4]. IPV prevalence and its effects were found to be worsened in post-conflict and resources limited setting like Rwanda due to low social support [5], burdened mental health problems [6], gender inequality and cultural and economic barriers [4, 7] that characterize these settings. Authors have revealed that IPV prevalence in Rwanda ranges from 16 to 50% and that it occurs in women and men even if women are more frequently and seriously affected [8, 9]. Rwanda is a patriarchal society, where IPV may be perceived as a confidential family matter and considered acceptable to keep the family together [8], despite gender-based violence (GBV) being widely recognized as a punishable offence in the Rwandan penal code. The stigma and discrimination that accompany victims of violence limit the ability of the Rwandan society to effectively address the GBV problem.

Violence against women was a central concern in this regard, and early studies on the relationship between violence against women and reproductive health in developing countries led to a greater understanding of the problem and its related adverse health outcomes [10]. Outcomes of IPV for pregnant women include pregnancy loss, HIV infection, stress, depression, pre-term delivery, abortion, stillbirth; as well as to giving birth to babies susceptible to low birth weight, illness, malnutrition and mortality [3, 11].

Women who experience IPV have myriad needs of services from different sectors, including antenatal care (ANC) service which is one of the best approaches to preventing poor pregnancy outcomes [1]. It was found that optimal ANC includes initiating care within the first 3 months of pregnancy, receiving at least four ANC visits and receiving care from skilled providers [12]. Antenatal care provides an opportunity to inform and educate pregnant women on important health issues including health promotion activities, screening and diagnosis [13]. It also offers an important platform to communicate with and support women, families and communities at a critical time in women’s life [14]. Thus, ANC is an essential component of birth preparedness complications readiness (BP/CR) matrix, a process of planning for normal birth and actions to be done in case of emergency. BP/CR matrix delineates “the roles of policymakers, facility managers, providers, communities, families, and women in ensuring that women and newborns receive appropriate, effective, and timely care” [4, 8]. Women and newborns need timely access to skilled care during pregnancy, childbirth and postpartum period [4, 8].

There has been an increase in the use of ANC services among pregnant women in low- and middle-income countries (LMICs) since 2002, but the evidence suggests that there remains a need to improve the quality of care that is given in ANC clinics and delivery [13]. According to the Demographic and Health Survey (DHS, 2014–2015) report, overall, four in ten women aged from 15 to 49 years reported exposure to “*emotional, physical or sexual violence*” from their intimate partners. Sexual violence committed by husband or partner was very common standing at 34 % and 20% of women aged 15–49 reported having exposed to sexual violence in their entire life. Evidence shows that women with at least five children are more likely to be exposed to physical violence (46%) than women with no children (23%) [15]. Mothers normally receive ANC services from health centers and district hospitals. Although severe cases are referred to the national referral hospitals, district hospitals have equipment and capacity to provide services related to ANC [14]. Authors have indicated that 99 % of Rwandan women received ANC from skillful health care providers (doctor, nurse, medical assistant, and midwife) [15], but the frequency of visits was lower than the standard set by World Health Organization (WHO) and the Rwandan Ministry of Health. In fact, only 44 % of mothers had at least four ANC visits. This proportion represents an increase from 13% in 2005 and 35% in 2010. Noteworthily, 56 % of women who went to their first ANC visit during the 1st trimester of pregnancy, as recommended by WHO [15].

Previous studies have documented the prevalence and correlates of IPV in large parts of the world based on different characteristics of a population, prevalence, and association of IPV with reproductive health issues [16]. IPV studies in developing countries mainly relied on data from their nationally representative Demographic and Health Surveys (DHS) [17]. Despite a pool of literature on the association of IPV exposure with low ANC services utilization in high income countries (e.g. USA [18], India [19], Honduras [12] and etc.) and few in developing countries (e.g. Nigeria [16], Mozambique [20], Ethiopia [21], Nepal [22] and etc.), there is a dearth of studies on the specific IPV exposure associated with reduced ANC services utilization especially in post-genocide countries. The primary goal of the current study is to analyze the effects of three specific IPV (i.e. physical IPV, emotional and sexual IPV) on ANC services utilization in Rwanda as post-genocide country. We hypothesized associations of exposure to emotional, physical and/or sexual IPV with ANC visits initiation within the first 3 months of pregnancy, having at least four ANC visits, and the receiving of care from skilled providers.

## Materials and methods

### Data source and sampling

Secondary data from the 2014–15 Rwanda Demographic Health Survey (RDHS 2014–15) was used in this sample. The RDHS survey was conducted by the National Institute of Statistics of Rwanda (NISR) from November 9, 2014, to April 8, 2015, using a stratified two stage cluster design. The first stage involved selecting sample points (clusters) consisting of enumeration areas (EAs) delineated for the 2012 Rwanda Population and Housing Census. A total of 492 clusters were selected, 113 and 379 in urban and rural areas (respectively). At the second stage, a sample of households was drawn in each EA using a systematic sampling. Within each sample point 26 households were selected, for a total sample of 12,792 households. Approximately, 13,564 women aged 15–49 years were eligible to complete the questionnaire. Of 13,497 women aged 15–49 years who had completed the questionnaire during the survey, only 5116 women met the inclusion criteria for this study giving a response rate of 37.71%. The participants were eligible if they were married, living together with their husband/partner and had at least one child aged 0–5 years.

### Measures

#### Antenatal care service utilization

Antenatal Care services utilization was measured using three indicators: early ANC, sufficient ANC and skilled ANC providers. The early ANC is dichotomized into receiving early ANC services and delaying ANC services where ANC is considered to be early if a woman receives ANC visits within the first 3 months of pregnancy. Sufficient ANC is also evaluated as a dichotomized variable: receiving a sufficient ANC and receiving insufficient ANC where ANC is considered to be sufficient if a woman attended at least four ANC visits. Moreover, ANC providers were dichotomized into unskilled ANC providers (e.g. community health workers) and skilled providers (i.e. doctor, nurse, medical assistant and midwife).

#### Intimate partner violence

Intimate partner violence (IPV) was measured by items on physical (six items), sexual (four items) or emotional violence (two items) that were selected from the Women’s health and life experiences questionnaire, a validated questionnaire developed by the WHO for research on IPV experience [23]. This questionnaire was translated and back- translated into Kinyarwanda. This instrument has been shown to be cross-culturally valid [24–26] in both men and women. Sample items for physical violence subscale were: (“1). *Did your husband/partner ever kick you, beat you, drag you*.., (“2). *Did your husband/partner ever slap you*.. and etc.”; and Sexual abuse subscale included (“1). *Did your husband/partner ever physically forced you into unwanted sex..,* (“2)*. Did your husband/partner ever force you into other unwanted sexual acts* .. and etc. The emotional violence was assessed by the following two questions: (“1). *Have you ever been insulted or made to feel bad by our partner/husband*… and (“2). *Have you ever been humiliated by your partner/husband or have you ever been threatened with harm by your partner/husband*. Each item was scored on a dichotomous scale “yes” or “no”.

### Sociodemographic questionnaire

Women’s Health and Life Experiences questionnaire assessed the ages of both women and their husbands/partners as well as collected information on education level, women’s literacy (illiterate versus literate), types of residence, religion and women’s occupation status. We also asked about mass media exposure, sterilization and wealth index.

### Statistical analysis

Data was analyzed quantitatively using frequencies and percentages to describe the characteristics of ANC indicators, IPV and socio demographic factors (e.g., education level, residence, religion, occupation status, mass media exposure, province, age, parity and household size). For bivariate analysis, the Chi-Square test was utilized to examine the association in ANC services utilization indicators and forms of IPV via socio-demographic factors. In a multivariate analysis, after controlling for covariate factors, multiple logistic regression was used to examine whether emotional, physical or sexual IPV were associated with early ANC initiation, sufficient ANC and skilled ANC providers Where IPV was dichotomous coded variable (i.e. no = 0 and yes =1), No = 0 was chosen as a reference category in our multiple logistic regression. The covariates were chosen based on a purposeful selection process begun by a univariate analysis of each variable. Covariates were included based on results from Wald tests; those with *p*-values below .25 were retained [27]. All measures were presented as odd ratios (ORs), whereby an odd ratio greater than 1 is a positive association and an odd ratio less than 1 is negative association, with their 95% confidence intervals, and an odd ratio greater than 1 is a positive association and an odd ratio less than 1 is negative association, and fell within the 95% confidence interval. The Evaluation of the patterns of missing data indicated that data were missing completely at random with overall missing values of 3%. This rate was within the acceptable range (less than 5%) and it was replaced by median substitution as recommended by several researchers [28, 29]. However, the preliminary analysis illustrated no impact of missing data on the results of this study when they were replaced or not. All analyses were performed using the Statistical Package of Social Science (IBM SPSS Statistics 25).

## Results

**Fig. 1:**
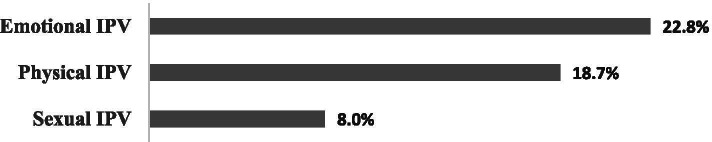
Forms of Intimate Partner Violence (%)

Most of sampled women were in rural areas (78.4%) and had a primary education level (70.9%) comparable to their husband’s primary education level (70.4%). The sample consisted largely of literate women (77.1%) and nearly all (99.5%) had a religion. Most of the sampled women were exposed to at least one type of mass media (86.4%) (Table 1). Regarding residential locations of the participants, 24.4% came from Eastern, 24.1% came from Western, and 23.8% came from Southern provinces, whereas, 15.8% come from Northern provinces with the remaining 11.9% coming from Kigali City. The mean parity was 4.46, with an average age of 31.59 years (Table 1). In the view of the IPV exposure, the majority experienced emotional (22.8%); 18.7% of all participants experienced physical violence; and only 8% of the sample reported experiencing sexual violence. The mean household size was 5.15 (Fig. 1).

**Table 1:** Distribution of variables considered in the analysis of relationship between IPV and ANC service utilization

Variables	Frequency	Percent
**Women’s education**		
No education	**785**	**15.4**
Primary	**3628**	**70.9**
Secondary	**542**	**10.6**
Higher	**161**	**3.1**
**Husband’s education**		
No education	**830**	**16.3**
Primary	**3597**	**70.4**
Secondary	**467**	**9.1**
Higher	**212**	**4.1**
**Women’s literacy**		
Not able to read at all	**1168**	**22.9**
Able to read a part/whole sentence	**3938**	**77.1**
**Type of place of residence**		
Urban	**1105**	**21.6**
Rural	**4011**	**78.4**
**Women’s religion**		
No religion or other	**24**	**0.5**
Has a religion	**5085**	**99.5**
**Women’s occupation**		
Not working/ don’t work	**305**	**6**
Working	**4811**	**94**
**Exposure to mass media**		
No type of mass media	**698**	**13.6**
One type of mass media	**2179**	**42.6**
Two types of mass media	**1432**	**28**
Three types of mass media	**780**	**15.2**
**Province**		
Kigali city	**608**	**11.9**
South	**1218**	**23.8**
West	**1232**	**24.1**
North	**810**	**15.8**
East	**1248**	**24.4**
Women’s age **(Mean)**	**31.59**	
Husband’s age **(Mean)**	**36**	
Wealth index **(mean)**	**2.96**	
Parity **(Mean)**	**4.45**	
Household’s size **(Mean)**	**5.15**	
***N*** **= 5116**

**Fig. 2:**
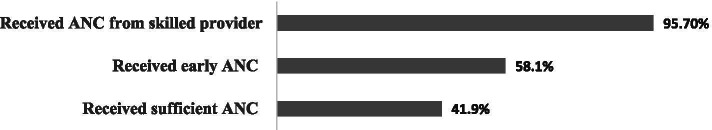
Antenatal Care Service Utilization Indicators (%)

In the current sample, the majority received ANC from skilled providers (97.01%), 58.7% received early ANC, and less than a half received at least four ANC visits during their most recent pregnancy (45.8%), (Fig. 2).

In model adjusting, we found a significant relationship between physical IPV and early ANC utilization and a sufficient number of ANC visits. There was no significant relationship between skilled ANC providers and IPV. While sexual IPV were not significant in all the three models, women who had ever experienced physical violence by their partners during the preceding 12 months were less likely to receive more than four ANC visits, (O.R = 0.6151, CI = 0.417–0.908) and they were less likely to attend the initial ANC visits within the first 3 months (O.R = 0.656, CI = 0.445–0.967), (Table 2).

**Table 2:** Adjusted odds ratios (aOR) and 95% confidence intervals (CIs) for associations between intimate partner violence (IPV) and antenatal care (ANC) among married women living together with partners with at least one child aged 5 years or younger, selected from Rwanda DHS 2014–15

	Early ANC	Sufficient ANC	Skilled ANC providers
	Adjusted OR(95% CI)	Adjusted OR(95% CI)	Adjusted OR(95% CI)
	*n* = 4583	*n* = 4612	*n* = 4574
Ever experienced emotional IPV			
No	1	1	1
Yes	0.945(0.656–1.360)	0.967(0.674–1.389)	1.073(0.348–3.306)
Ever experienced sexual IPV			
No	1	1	1
Yes	1.068(0.653–1.746)	0.931(0.568–1.527)	0.500(0.162–1.550)
Ever experienced physical IPV			
No	1	1	1
Yes	0.638(0.433–0.940)*	0.611(0.414–0.901)*	3.924(0.470–32.756)
Woman’s education			
No education	0.428(0.100–1.8410	0.327(0.089–1.196)	4.507(0.424–47.868)
Primary	0.456(0.116–1.791)	0.343(0.103–1.142)	3.080(0.236–40.174)
Secondary	0.653(0.173–2.464)	0.367(0.115–1.165)	na
Higher	1	1	
Husband’s education level			na
No education	0.576(0.196–1.709)	0.767(0.290–2.030)	
Primary	0.669(0.237–1.894)	0.890(0.354–2.238)	
Secondary	1.039(0.357–3.025)	0.837(0.327–2.146)	
Higher	1	1	
Woman’s literacy			
Illiterate	1.097(0.712–1.689)	1.031(0.676–1.572)	0.175(0.017–1.836)
Literate	1	1	1
Area of residence			na
Urban	0.828(0.546–1.256)	0.942(0.662–1.343)	
Rural	1	1	
Province			
Kigali city	0.469(0.284–0.775)*	1.271(0.775–2.086)	1.480(0.263–8.338)
South	1.174(0.815–1.690)	2.331(1.626–3.342)*	1.101(0.285–4.259)
West	0.909(0.639–1.293)	1.450(1.027–2.049)*	0.211(0.075–0.593)*
North	1.109(0.744–1.653)	1.607(1.091–2.368)*	2.998(0.343–26.174)
East	1	1	1
Exposed to mass media	na		na
No type		1.119(0.684–1.833)	
One types		1.097(0.733–1.643)	
Two types		1.123(0.741–1.702)	
Three types		1	
Woman’s current age	1.000(0.968–1.032)	1.002(0.971–1.035)	na
Husband/partner’s age	0.984(0.965–1.004)	0.983(0.964–1.004)	na
Wealth index		na	
Poorest	0.667(0.383–1.161)		3.488(0.824–14.764)
Poorer	0.631(0.368–1.080)		1.918(0.548–6.719)
Middle	0.662(0.388–1.127)		3.577(0.793–16.128)
Richer	0.606(0.364–1.009)		0.815(0.267–2.487)
Richest	1		1
Household’s size	0.865(0.784–0.954)*		1.270(0.987–1.635)

Source: Table prepared by the author based on the analyses

Models were adjusted for woman’s age, husband’s age, woman’s education, husband’s education, woman’s literacy, area of residence, religion, women’s occupation, wealth index, household size, women’s mass media exposure and province

*na* Not applicable

*Pvalue < 0.05

Although, most of the covariates used are not statistically significant in our model (Table 2), according to our bivariate analysis (Table 3), covariates like: women’s and husband’s education level, wealth index, mass media exposure and types of residence were significantly associated with IPV (Table 4).

**Table 3:** Socio demographic characteristics and antenatal care service utilization by married women living with partner and had at least one aged 5 years or under, selected from Rwanda DHS 2014–15

Characteristics	Early ANC		Sufficient ANC		Skilled ANC providers	
	Yes	No	Yes	No	Yes	No
	n (%)	n (%)	n (%)	n (%)	n(%)	n(%)
**Women’s education**						
No education	324(12.2)	329(17.2)	256(12.10	406(16.3)	644(14.4)	8(7.8)
Primary	1875(70.5)	1411(73.6)	1500(71.1)	1803(72.2)	3196(71.5)	83(81.4)
Secondary	346(13)	152(7.9)	246(11.7)	254(10.2)	488(10.9)	10(9.8)
Higher	116(4.4)	26(1.4)	109(5.2)	34(1.4)	140(3.1)	1(1.0)
*P* value	0.00*		0.00*		**0.113**	
**Husband’s education**						
No education	362(13.6)	350(18.3)	293(13.9)	423(17)	13(12.7)	699(15.7)
Primary	1874(70.6)	1378(72.0)	1489(70.7)	1786(71.6)	76(74.5)	3168(71.1)
Secondary	267(10.1)	147(7.7)	201(9.5)	214(8.6)	10(9.8)	403(9.0)
Higher	152(5.7)	39(2.0)	122(5.8)	70(2.8)	3(2.9)	188(4.2)
*P* value	0.00*		0.00*		**0.848**	
**Women’s literacy**						
Not able to read at all	548(20.6)	454(23.8)	435(20.7)	579(23.2)	983(21)	16(15.7)
Able to read	2110(79.4)	1457(76.2)	1671(79.3)	1913(76.8)	86(84.3)	86(84.3)
*P* value	0.011*		0.036*		**0.124**	
**Type of place of residence**						
Urban	596(22.4)	382(19.9)	468(22.2)	518(20.7)	955(21.4)	21(20.6)
Rural	2065(77.6)	1536(80.1)	1643(77.8)	1979(79.3)	3513(78.6)	81(79.4)
*P* value	0.043*		**0.24**		**0.848**	
**Women’s religion**						
No religion	8(0.3)	8(0.4)	13(0.5)	5(0.2)	16(0.4)	0(0.0)
Has religion	2651(99.7)	1907(99.5)	2481(99.5)	2104(99.8)	4447(99.6)	102(100)
*P* value	**0.782**		**0.304**		**0.813**	
**Women’s occupation**						
Not working/ don’t work	126(6.6)	161(6.1)	120(5.7)	168(6.7)	5(4.9)	282(6.3)
Working	1792(93.4)	2500(93.9)	1991(94.3)	2329(93.3)	97(95.1)	4186(93.7)
*P* value	**0.475**		**0.145**		**0.562**	
**Province**						
Kigali city	279(10.5)	257(13.4)	223(10.6)	317(12.7)	526(11.8)	8(7.8)
South	677(25.4)	403(21)	572(27.1)	515(20.6)	1070(23.9)	9(8.8)
West	624(23.4)	495(25.8)	526(24.9)	604(24.2)	1049(23.5)	68(66.7)
North	445(16.7)	274(14.3)	351(16.6)	370(14.8)	711(15.9)	5(4.9)
East	636(23.9)	489(25.5)	439(20.8)	691(27.7)	1112(24.9)	12(11.8)
*P* value	0.00*		0.00*		0.00*	
**Exposed to mass media**						
No type	350(13.2)	279(14.6)	271(12.9)	365(14.7)	616(13.9)	12(11.9)
One type	1139(43)	825(43.3)	899(42.7)	1078(43.5)	1911(43.0)	51(50.5)
Two types	745(28.1)	524(27.5)	582(27.7)	690(27.8)	1237(27.8)	27(26.7)
Three types	414(15.6)	279(14.6)	351(16.7)	348(14.0)	681(15.3)	11(10.9)
*P* value	**0.471**		0.043*		**0.41**	
**Women’s age** (Mean)	30.29	31.79	30.59	31.2	30.92	30.8
*P* value	0.00*		**0.199**		**0.885**	
**Husband’s age** (Mean)	34.48	36.32	34.78	35.67	35.27	34.17
*P* value	0.00*		**0.063**		**0.971**	
**Wealth index** (mean)	2.99	2.88	2.99	2.91	2.95	2.89
*P* value	0.04*		**0.437**		**0.183**	
**Parity** (Mean)	4.24	4.65	4.32	4.52	4.41	5
*P* value	**0.375**		**0.133**		**0.862**	
**Household’s size** (Mean)	4.86	5.35	4.91	5.2	5.06	5.07
*P* value	0.00*		0.00*		**0.904**	

Source: Table prepared by the authors based on their analyses

*P-value < 0.05

**Table 4:** Socio demographic characteristics and Intimate Partner Violence by married women living with partner and had at least one aged 5 years or under, selected from Rwanda DHS 2014–15

Characteristics	Any physical IPV		Any sexual IPV		Any emotional IPV	
	Yes	No	Yes	No	Yes	No
	n (%)	n (%)	n (%)	n (%)	n(%)	n(%)
**Women’s education**						
No education	31(13.7)	147(14.9)	17(17.3)	161(14.3)	42(15.1)	135(14.3)
Primary	184(81.1)	714(72.2)	71(72.4)	833(74.1)	221(79.2)	685(72.5)
Secondary	10(4.4)	108(10.9)	8(8.2)	110(9.8)	14(5.0)	105(11.1)
Higher	2(0.9)	20(2.0)	2(2.0)	20(1.8)	2(0.7)	20(2.1)
*P* value	0.009*		**0.83**		0.007*	
**Husband’s education**						
No education	47(20.8)	147(14.9)	15(15.3)	180(16.1)	65(23.4)	130(13.8)
Primary	167(73.9)	716(72.6)	75(76.5)	813(72.6)	195(70.1)	694(73.7)
Secondary	10(4.4)	88(8.9)	7(7.1)	92(8.2)	18(6.5)	81(8.6)
Higher	2(0.9)	35(3.5)	1(1.0)	35(3.1)	0(0.0)	37(3.9)
*P* value	0.005*		**0.741**		0.00*	
**Women’s literacy**						
Not able to read at all	54(23.9)	225(22.8)	23(23.5)	256(22.9)	65(23.3)	214(22.7)
Able to read	172(76.1)	761(77.2)	75(76.5)	864(77.1)	214(76.7)	727(77.3)
*P* value	**0.729**		**0.89**		**0.846**	
**Type of place of residence**						
Urban	30(13.2)	201(20.3)	18(18.4)	215(19.1)	45(16.1)	189(20.0)
Rural	197(86.8)	788(79.7)	80(81.6)	909(80.9)	234(83.9)	756(80.0)
*P* value	0.014*		**0.854**		**0.149**	
**Women’s religion**						
No religion	0(0.0)	2(0.2)	0(0)	2(0.2)	1(0.4)	1(0.1)
Has religion	227(100.0)	986(99.8)	98(100)	1121(99.8)	278(99.6)	943(99.9)
*P* value	**0.708**		**0.877**		**0.566**	
**Women’s occupation**						
Not working/ don’t work	7(3.4)	68(6.7)	6(6.1)	68(6.0)	14(5.0)	61(6.5)
Working	200(96.6)	946(93.6)	92(93.9)	1056(92.0)	265(95.0)	884(93.5)
*P* value	**0.24**		**0.977**		**0.397**	
**Exposed to mass media**						
No type	45(19.9)	131(13.3)	19(19.6)	158(14.1)	49(17.7)	27(13.5)
One type	100(44.2)	442(45.0)	41(42.3)	502(44.9)	120(43.3)	425(45.2)
Two types	53(23.5)	266(27.1)	26(26.8)	295(26.4)	75(27.1)	247(26.3)
Three types	28(12.4)	144(14.6)	11(11.3)	163(14.6)	33(11.9)	41(15.0)
***P*value**	0.007*		**0.454**		**0.237**	
**Women’s age** (Mean)	1.07		31.46	30.77	31.75	30.54
***P*value**	**0.961**		**0.709**		0.004*	
**Husband’s age** (Mean)	35.01	35.04	35.57	34.99	36.66	34.56
***P*value**	**0.356**		**0.575**		0.007*	
**Wealth index** (mean**)**	2.55	3.01	2.76	2.94	2.68	2.99
***P*value**	0.00*		**0.465**		0.022*	
**Parity** (Mean**)**	4.5	4.22	5	4.88	4.38	4.2
***P*value**	**0.786**		**0.103**		**0.146**	
**Household’s size** (Mean)	4.83	4.91	5.02	5.15	5.05	4.84
***P*value**	**0.802**		**0.819**		**0.712**	

Source: Table prepared by the author based on their analyses

*Pvalue < 0.05

## Discussion

To the best of our knowledge, this is the first study to investigate the relationship between different types of IPV (i.e. physical, sexual and emotional violence) and ANC services utilization in Rwanda. As predicted, we found that there was a significant negative association of physical IPV with both early ANC and sufficient ANC. Regarding the ANC services utilization, this study found that 97.7% of the current sample had received ANC services from skilled providers. Further, 46 % received at least four ANC visits during pregnancy time and 58.1% received early ANC. The prevalence of attending at least four or more ANC visits appears to be lower in Rwanda than those reported in some other East African countries like Zimbabwe (75.7%), Uganda (60%), Kenya (57.6%) and Zambia (56%) [30]. The difference observed could be due to post-genocide situation of Rwanda in terms of low social support [4] and elevated mental health problems [31, 32]. This undeniably life-altering event left many women without parents, siblings, external relatives and friends (i.e., their supportive network) for they have perished. Resultantly, expecting mothers may not have someone who physically lives at home with them when they have to go to seek ANC services. With the role of support from significant others or family members embodying understanding, sympathy and communication, which may positively affect ANC services attendance. Furthermore, throughout the region, high rates of Posttraumatic stress disorder (PTSD), depression, and other psychological disorders have been observed following the 1994 genocide perpetrated against Tutsi [26, 33] which in turn lead to high risks of violence committed against women [6, 34] and poor ANC utilization [31, 32].

Similar to other studies using large samples, we found a greater prevalence of emotional victimization (22.8%) than physical (18.7%) and sexual (8%) victimizations among our sample of pregnant women. A study conducted in Uganda found that the rate of emotional violence (40%) was higher than sexual violence (23%) in married women [35]. A greater prevalence of emotional violence (29%) than physical victimization (6%) was also found in a sample of American pregnant women [36]. Despite observing higher percentages, the prevalence of different types of IPV reported in this study were still smaller than those found in many community-based samples. It was found that the rate of physical IPV in women was 37% in Kenya [], 41% in Uganda [35], 38% in Egypt [12], 48% in Bangladeshi and 48.8% in Zambia [12]. They further indicated that in sub-Saharan Africa the prevalence of emotional IPV in women ranged from 7% in Comoros to 40.1% in Cameroon, while sexual IPV ranged from 3% in Moldova to 26% in Bangladesh [12].

Despite these differences, our results indicate an alarming amount of emotional victimization experienced by pregnant women, which may be explained by spousal age difference. The tradition of male dominance in marriage, which remains prevalent in Rwanda, may be attributed to: men having a strong preference to marry women younger than themselves or the common attempt by men in creating an avenue for men to exercise power as the family head [38]. Marriages with substantial age gap may lead to emotional violence due to differences in maturity and differences in opinions [39]. Worryingly, different authors found that emotional victimization was significantly associated with poorer maternal health and depression, and child’s health and temperament [36].

As predicted and similar to prior studies, the bivariate analysis revealed that socio-demographic characteristics such as age of women, rural residence [40, 41], women and husband’s education level [42] were significantly associated with early ANC or sufficient ANC. The current study demonstrates that the education and economic level of most women and their husbands were very low although husbands seemed to have a better status than wives in these aspects. In contrast, authors have found that there is no significant association between early ANC and women’s education [40, 41]. Another study assessing the factors related to utilization of ANC services in Vietnam demonstrated that age did not affect its utilization [43]. Our results could be explained by a greater self-efficacy gained from the experience of prior pregnancies among upper aged women; therefore, the ones who give birth to their first child without any complication are more likely to make insufficient ANC visits during subsequent pregnancies [44].

Moreover, Saad-Haddad et al. has found an association between women’s education level and having a higher frequency (> 4) of ANC visits [45]. Different authors have highlighted that women in the higher age group who are either single, divorced, separated or widowed women were at a higher risk of making two or less visits to ANC services [4]. Furthermore, women with poor social networks (e.g. no significant others, or close friends to assist when in need) were found to be at risk of poor utilization of ANC services. However, school attendance or household assets, as a proxy for socioeconomic status, were not found to be associated with poor utilization of ANC services [4]. Further investigations should consider other additional socio-demographic factors including awareness of ANC services, distance between home and health facility and planned Pregnancy (as opposed to an unplanned pregnancy) and age at first visit.

The bivariate analysis also revealed that women’s and husband’s low education level, rural residence, low exposure to mass media and poor wealth index were associated with physical IPV. Similar to our findings, substantial body of literature has indicated that woman’s and husband’s low education levels, poor wealth index [46, 47], rural residence and low exposure to mass media were and live in a rural residential setting are contributing factors associated with physical IPV [12]. Frequent exposure to mass media seemed to increase women’s exposure to IPV in the current sample. In support of this contention, a study conducted in Bangladesh showed that frequent exposure to newspaper, magazine or television was negatively connected with justifying IPV; while exposure to radio was positively connected [48].

Unexpectedly, our findings demonstrated that all socio-demographic characteristics of women and their partners were not associated with sexual IPV. However, our findings highlighted that only low education levels of women and their husband were significantly linked to emotional IPV when all socio-demographic characteristics were considered. Inconsistently, prior studies indicated that older age and poor wealth index were associated with emotional IPV [38, 49, 50]. Although physical abuse is suggested to diminish with age, prevalence of emotional abuse appears to be constant over the lifetime [38].

Even though several studies have found a significant relationship between physical IPV and receiving services from skilled ANC providers [46, 51], our model which was adjusted for covariates failed to find a significant association between the two (physical IPV and receiving skilled ANC providers).

Different scholars found that physical IPV was significantly related to sufficient ANC and early ANC [51, 52] which is in congruence with our findings. However, our present study is best supported with investigation conducted by Koski et al. which indicated that women who experienced physical IPV were less likely to initiate early ANC and less likely to receive four or more than four ANC visits consistent to our present study [46, 53].

### Limitations

Although Rwanda Demographic Health Survey (DHS) dataset is well documented, well known and tested by researchers, during our analysis, we found that some of the attributes were not available i.e. the employment status of respondents which would’ve been an important covariates in the sample. There were also limitations in accessing similar studies on IPV violence and ANC services utilization that are based in Rwanda-- which only further revealed the gap that exists in the literature review. Furthermore, some attributes had missing values which can have a significant effect on drawing conclusion; however, the overall missing values of 3% reported in the current study was in the acceptable range (less than 5%) and was replaced by median substitution [28, 29]. The last limitation of this study was measurement limitation i.e. variables of interest were dichotomized which prevented an evaluation of graded effects of IPV on ANC.

## Conclusion

The findings of the present study indicate a significant association between physical intimate partner violence (IPV) and antenatal care services utilization indicators of early ANC service and sufficient ANC. Emotional and sexual IPV were not significant after controlling for several socio-demographic characteristics (i.e. women’s educational level, partner’s education level, women’s literacy, residence, mass media exposure, religion, age, household size, wealth index and province); though most of them were not statistically significant as well. Therefore, this analysis provides support for continued efforts to reduce intimate partner violence, through the improvement of screening for IPV. These results also call attention to the need for greater access to ANC services, especially for women with the characteristics of this sample (primarily rural, East-African, and residing in a post-genocide country).

## Data Availability

The datasets used and/or analysed during the current study are available from the corresponding author on reasonable request.

## References

[1] WHO & Pan American Health Organization. Understanding and addressing violence against women: intimate partner violence. Geneva: World Health Organization; 2012. https://apps.who.int/iris/handle/10665/77432.

[2] WHO (2013). Violance against women:global picture health response.

[3] Isaac O, Harryson K, Margaret A (2017). Intimate partner violence in pregnancy among antenatal attendees at health facilities in West Pokot county, Kenya.

[4] Rurangirwa A, Mogren I, Ntaganira J, Krantz G (2017). Intimate partner violence among pregnant women in Rwanda, its associated risk factors and relationship to ANC services attendance: a population-based study.

[5] Dougé N, Lehman EB, McCall-Hosenfeld JS (2014). Social support and employment status modify the effect of intimate partner violence on depression symptom severity in women: results from the 2006 behavioral risk factor surveillance system survey. Womens Health Issues.

[6] Yu R, Nevado-Holgado AJ, Molero Y, D'Onofrio BM, Larsson H, Howard LM, Fazel S (2019). Mental disorders and intimate partner violence perpetrated by men towards women: a Swedish population-based longitudinal study. PLoS Med.

[7] Small M, Gupta J, Frederic R, Joseph G, Theodore M, Kershaw TI (2008). Intimate partner and nonpartner violence against pregnant women in rural Haiti. Int J Gynaecol Obstet.

[8] Slegh H, Kimonyo A (2010). Masculinity and gender based violence in Rwanda: experiences and perceptions of men and women.

[9] Mukashema IA (2017). Report about intimate partner violence in southern and western Rwanda. Int J Child Youth Fam Stud.

[10] Kishor S, Johnson K. Profiling domestic violence: a multi-country study. Stud Fam Plan. 2005;36(3):259+. https://link.gale.com/apps/doc/A137718265/AONE?u=anon~504ec891&sid=googleScholar&xid=0f0ff522.

[11] Umubyeyi A, Mogren I, Ntaganira J, Krantz G (2014). Intimate partner violence and its contribution to mental disorders in men and women in the post genocide Rwanda: findings from a population based study. BMC Psychiatry.

[12] Sebert Kuhlmann A, Foggia J, Fu Q, Sierra M (2017). Intimate partner violence as a predictor of antenatal care service utilization in Honduras. Rev Panam Salud Publica.

[13] Rurangirwa A, Mogren I, Ntaganira J, Govender K, Krantz G (2018). Quality of antenatal care services in Rwanda: assessing practices of health care providers. BMC Health Serv Res.

[14] Rurangirwa A, Mogren I, Ntaganira J, Govender K, Krantz G (2018). Quality of antenatal care services in Rwanda: assessing practices of health care providers.

[15] NISR (2016). Rwanda demographic and health survey 2014–15.

[16] Lukman SB (2014). Association between intimate partner violence and utilisation of maternal health services in Nigeria.

[17] Gomez AM (2011). Sexual violence as a predictor of unintended pregnancy, contraceptive use and unmet need among female youth in Colombia. J Women's Health.

[18] Masho SW, Rozario SS, Ferrance JL (2019). Intimate partner violence around the time of pregnancy and utilization of WIC services. Matern Child Health J.

[19] Paul P, Mondal D (2021). Investigating the relationship between women’s experience of intimate partner violence and utilization of maternal healthcare services in India. Sci Rep.

[20] Tura H, Licoze A (2019). Women’s experience of intimate partner violence and uptake of Antenatal Care in Sofala, Mozambique. PLoS One.

[21] Gashaw BT, Magnus JH, Schei B (2019). Intimate partner violence and late entry into antenatal care in Ethiopia. Women Birth.

[22] Singh JK, Evans-Lacko S, Acharya D, Kadel R, Gautam S (2018). Intimate partner violence during pregnancy and use of antenatal care among rural women in southern Terai of Nepal. Women Birth.

[23] Ellsberg M, Heise L (2005). Researching violence against women: a practical guide for researchers and activists.

[24] Nybergh L, Taft C, Krantz G (2012). Psychometric properties of the WHO Violence Against Women instrument in a male population-based sample in Sweden. BMJ Open.

[25] Nybergh L, Taft C, Krantz G (2013). Psychometric properties of the WHO Violence Against Women instrument in a female population-based sample in Sweden: a cross-sectional survey. BMJ Open.

[26] Rubanzana W, Ntaganira J, Freeman MD, Hedt-Gauthier BL (2015). Risk factors for homicide victimization in post-genocide Rwanda: a population -based case- control study. BMC Public Health.

[27] Bursac Z, Gauss CH, Williams DK, Hosmer DW (2008). Purposeful selection of variables in logistic regression. Source Code Biol Med.

[28] Tabachnick BG, Fidell LS (2007). Using multivariate statistics.

[29] Raymond MR (1986). Missing data in evaluation research. Eval Health Prof.

[30] Tessema ZT, Minyihun A (2021). Utilization and determinants of antenatal care visits in east African countries: a multicountry analysis of demographic and health Surveys. Adv Public Health.

[31] Bitew T, Hanlon C, Kebede E, Medhin G, Fekadu A (2016). Antenatal depressive symptoms and maternal health care utilisation: a population-based study of pregnant women in Ethiopia. BMC Pregnancy Childbirth.

[32] Kim ET, Ali M, Adam H, Abubakr-Bibilazu S, Gallis JA, Lillie M, Hembling J, McEwan E, Baumgartner JN. The effects of antenatal depression and women’s perception of having poor health on maternal health service utilization in northern Ghana. Matern Child Health J. 2021;25(11):1697–706. 10.1007/s10995-021-03216-1. 10.1007/s10995-021-03216-1 34405361

[33] Schaal S, Weierstall R, Dusingizemungu J (2012). Mental health 15 years after the killings in Rwanda: imprisoned perpetrators of the genocide against the Tutsi versus a community sample of survivors. J Trauma Stress.

[34] Shorey RC, Fite PJ, Menon SV, Cohen JR, Stuart GL, Temple JR (2021). The association between PTSD symptoms and IPV perpetration across 6 years. J Interpers Violence.

[35] Gubi D, Nansubuga E, Wandera (2020). Correlates of intimate partner violence among married women in Uganda: a cross-sectional survey.

[36] McMahon S, Huang C-C, Boxer P, Postmus JL (2011). The impact of emotional and physical violence during pregnancy on maternal and child health at one year post-partum. Child Youth Serv Rev.

[37] Muriithi JKN, Ngige LW, Kimani E. Forms and prevalence of intimate partner violence experienced by women survivors in shelter homes in Kenya. Int J Human Soc Sci. 2017;7(3):2221-0989.

[38] Adebowale AS (2018). Spousal age difference and associated predictors of intimate partner violence in Nigeria. Adebowale BMC Public Health.

[39] Adebowale A (2018). Spousal age difference and associated predictors of intimate partner violence in Nigeria. BMC Public Health.

[40] Zegeye A, Bitew B, Koye D (2013). Prevalence and determinants of early antenatal care visit among pregnant women attending antenatal care in Debre Berhan Health Institutions, Central Ethiopia. Afr J Reprod Health.

[41] Geta MB, Yallew W. Early initiation of antenatal care and factors associated with early antenatal care initiation at health facilities in southern Ethiopia. Adv Public Health. 2017;2017:6

[42] Ahinkorah BO, Ameyaw EK, Seidu AA, Odusina EK, Keetile M, Yaya S (2021). Examining barriers to healthcare access and utilization of antenatal care services: evidence from demographic health surveys in sub-Saharan Africa. BMC Health Serv Res.

[43] Swenson IE, Thang NM, Nhan VQ, Tieu PX (1993). Factors related to the utilization of prenatal care in Vietnam. J Trop Med Hyg.

[44] Srivastava A, Mahmood S, Mishra P, Shrotriya VP (2014). Correlates of maternal health care utilization in Rohilkhand region, India. Ann Med Health Sci Res.

[45] Saad-Haddad G, DeJong J, Terreri N, Restrepo-Méndez MC, Perin J, Vaz L, Newby H, Amouzou A, Barros AJ, Bryce J (2016). Patterns and determinants of antenatal care utilization: analysis of national survey data in seven countdown countries. J Glob Health.

[46] Rahman M, Nakamura K, Seino K, Kizuki M (2012). Intimate partner violence and use of reproductive health services among married women: evidence from a national Bangladeshi sample. BMC Public Health.

[47] Vung N, Ostergren P, Gunilla KG (2008). Intimate partner violence against women in rural Vietnam -different socio-demographic factors are associated with different forms of violence: need for new intervention guidelines?. BMC Public Health.

[48] Krause KH, Haardörfer R, Yount KM (2017). Individual schooling and women’s community-level media exposure: a multilevel analysis of normative influences associated with women’s justification of wife beating in Bangladesh. J Epidemiol Community Health.

[49] Kishor S, Johnson K (2004). Profiling domestic violence – a multi-country study.

[50] Ghimirea D, Axinnb W, Smith-Greenaway E (2015). Impact of the spread of mass education on married women’ experience with domestic violence. Soc Sci Res.

[51] Hindin MJ, Sunita K, Donna LA (2008). Intimate partner violence among couples in 10 DHS countries: predictors and health outcomes.

[52] Leight J, Wilson N (2021). Intimate partner violence and maternal health services utilization: evidence from 36 national household surveys. BMC Public Health.

[53] Koski AD, Stephenson R, Koenig MR (2011). Physical violence by partner during pregnancy and use of prenatal care in Rural India. J Health Popul Nutr.

